# Assessing Connectivity Thresholds Under Habitat Loss Scenarios for Threatened Amphibians and Squamate Reptiles in the Eastern Brazilian Amazon

**DOI:** 10.1002/ece3.71741

**Published:** 2025-07-15

**Authors:** Cássia Teixeira, Gisele Lopes Nunes, Leonardo Carreira Trevelin, Daniel Paiva Silva, Ana Lúcia da Costa Prudente

**Affiliations:** ^1^ Programa de Pós‐Graduação em Biodiversidade e Evolução Museu Paraense Emílio Goeldi Belém Brazil; ^2^ Instituto Tecnológico Vale—Desenvolvimento Sustentável Belém Brazil; ^3^ COBIMA Lab, Departamento de Biologia Instituto Federal Goiano—Campos Urutaí Goiás Brazil

**Keywords:** ecological corridors, extinction threshold, landscape change, species distribution models

## Abstract

The extinction threshold hypothesis proposes a minimum of 30% habitat in a landscape to prevent isolation from affecting populations to local extinction. In this study, we tested scenarios of habitat loss in the landscape to evaluate whether the 30% habitat threshold is a good predictor of functional connectivity for 14 terrestrial herpetofauna species in eastern Brazilian Amazon landscapes. We evaluated functional connectivity across various habitat loss scenarios, utilizing species distribution models and landscape connectivity indices. We were able to demonstrate that below 32% habitat, overall regional connectivity in Southeastern Amazonia erodes, disturbing the ability of species to track environments within their climatic limits. However, species inhabiting montane savannahs in the region did not respond well to this 30% threshold and required the presence of sufficient areas to assess a possible decline. We also discovered that, when evaluated together, small patches in the landscape contributed to the integral connectivity of the study area and may demonstrate their importance as links between larger patches. Our results provide critical insights into the conservation needs of forest and montane savannah species, highlighting that while forest species adhere closely to a habitat threshold, montane savannah species require a different approach for conservation.

## Graphical abstract

We assessed which habitat extinction threshold in a landscape is a good predictor of functional connectivity for 14 species of terrestrial herpetofauna in the eastern Brazilian Amazon. We concluded that forest species should maintain a threshold of approximately 30% of habitat to avoid a significant abrupt decline in overall connectivity. However, species inhabiting montane savannas did not respond well to this threshold and required the presence of sufficient areas to evaluate a potential decline.

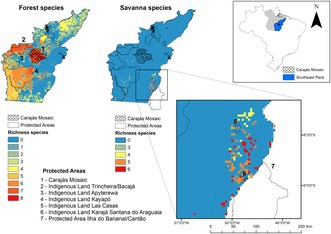

## 1 Introduction

In recent decades, anthropogenic climate changes and the reduction of natural habitats have been identified as major drivers of global biodiversity decline (Parmesan 2006; Haddad et al. 2015; Jaureguiberry et al. 2022). Considering the latest climate projections and rising deforestation rates, these factors are estimated to trigger considerable species loss globally (Tylianakis et al. 2008; Sampaio et al. 2018; IMAZON 2023). Climate change and habitat loss in the Amazon rainforest are predicted to synergistically reduce the potential distribution areas for several tree and thermoconforming lizard species in current and future scenarios (Gomes et al. 2019; Teixeira et al. 2022).

The Amazon rainforest plays a vital role in evapotranspiration, with atmospheric moisture sustaining regional hydrological cycles, regulating local precipitation and temperature, and influencing these patterns on a continental scale (Sampaio et al. 2018). Deforestation in the Amazon basin, driven by activities such as livestock farming, monocultures, and mining, increased by 29% in 2021, the highest in a decade (Dobrovolski et al. 2018; Soares‐Filho and Rajão 2018; IMAZON 2023). Exceeding a deforestation threshold could prevent rainforests from maintaining their climate, leading to extreme droughts and the transformation of forests into savannas (Lovejoy and Nobre 2018; Marengo et al. 2018). The primary consequence for biodiversity is the inability of species to geographically track environments within their climatic limits, which could eventually lead to extinction (Cowling et al. 2004; Feeley and Silman 2016; Gomes et al. 2019).

At the landscape scale, habitat loss reduces population sizes and increases susceptibility to stochastic effects, while habitat fragmentation divides remaining areas and isolates populations with limited dispersal capacity (Fahrig 1997; Fischer and Lindenmaye 2007). These processes interact non‐linearly, with simulation studies predicting a drastic decrease in natural population abundance when habitat is reduced below a specific limit, termed the “extinction threshold.” Although simulated to range from 10% to 30% among birds and mammals (Andrén 1994), this critical threshold of habitat in the landscape can vary considerably among species of other taxonomic groups depending on their habitat requirements and mobility (Luck 2005; Fahrig 2013), resulting, nonetheless, in populations of many species not being able to survive in small, isolated habitat patches (Fahrig 2003; Swift and Hannon 2010; Pardini et al. 2010). The extinction threshold hypothesis (ETH) posits the amount of habitat needed to maintain viable populations in anthropogenic landscapes (Arroyo‐Rodriguez et al. 2020). Highly fragmented landscapes hinder the dispersal of genes, propagules, and individuals, disrupting ecological processes and preventing species from tracking environments within their climatic limits (Cowling et al. 2004; Barlow et al. 2016; Feeley and Silman 2016).

A sensible strategy for addressing conservation in fragmented landscapes is using ecological corridors designed to maintain or restore connectivity between the remaining habitat patches (Rudnick et al. 2012). A practical, comprehensive approach to measuring this connectivity involves combining attributes of habitat patches (e.g., size, environmental suitability) with metrics quantifying pairwise connections between them (e.g., lowest cost, shortest distance; Saura and Torné 2009). This combination aims to assess the integral connectivity of a landscape, that is, its overall accessible habitat area (Saura and Torné 2009; Saura and Rubio 2010), by considering the dispersal limits and ecological characteristics of species and the physical structure of the landscape. Such a functional approach to connectivity has been employed to evaluate the effectiveness of protected areas acting as movement refuges for species (Tischendorf and Fahrig 2000; Laurance et al. 2012; Rudnick et al. 2012).

The southeastern Amazon basin encompasses deforestation frontiers, featuring landscapes with a long history of unsustainable human occupation (Fearnside 2005). The region's past and present climatic conditions, along with its geological attributes, created unique and diverse environments that support heterogeneous and endemic biodiversity that coexist with the main mining operations in Brazil (Martins et al. 2012). This region includes typical lowland rainforests and unique savanna forests on highland rocky outcrops, known as “*cangas*,” contributing to a heterogeneous biodiversity mosaic. Its terrestrial herpetofauna, for instance, comprises approximately 71 amphibian species and 120 squamate species, including some short‐range *canga* endemic species that are restricted to protected areas serving as refuges (Martins et al. 2012; Pinheiro et al. 2012).

In this study, we investigated patterns in the amount of habitat across landscapes to determine the minimum needed to maintain regional connectivity for Amazonian herpetofauna species. We specifically aimed for thresholds for the functional connectivity of these species in eastern Brazilian Amazon landscapes. We initially predicted that for this region forest species would have more connected potential habitat areas and, consequently, be less vulnerable to habitat loss than canga species.

Our main objective was to understand the mechanism resulting from the combined effects of loss of climatic suitability and habitat loss. To this end, we present a hierarchical methodology that integrates the two analytical scales. The first scale utilizes species distribution models (SDMs) for threatened herpetofauna to assess land use and land cover at the regional level in southeastern Pará. The second scale focuses on the landscape level, analyzing functional connectivity within the region. Species abundance and richness data are the most commonly used to assess habitat loss thresholds, but in this study, we propose the use of functional connectivity as an indirect variable to identify thresholds for local herpetofauna. With this, we seek to associate possible reductions in functional connectivity with the availability of habitats of high environmental suitability and discuss population declines.

## 2 Material and Methods

### 2.1 Study Region

**FIGURE 1:**
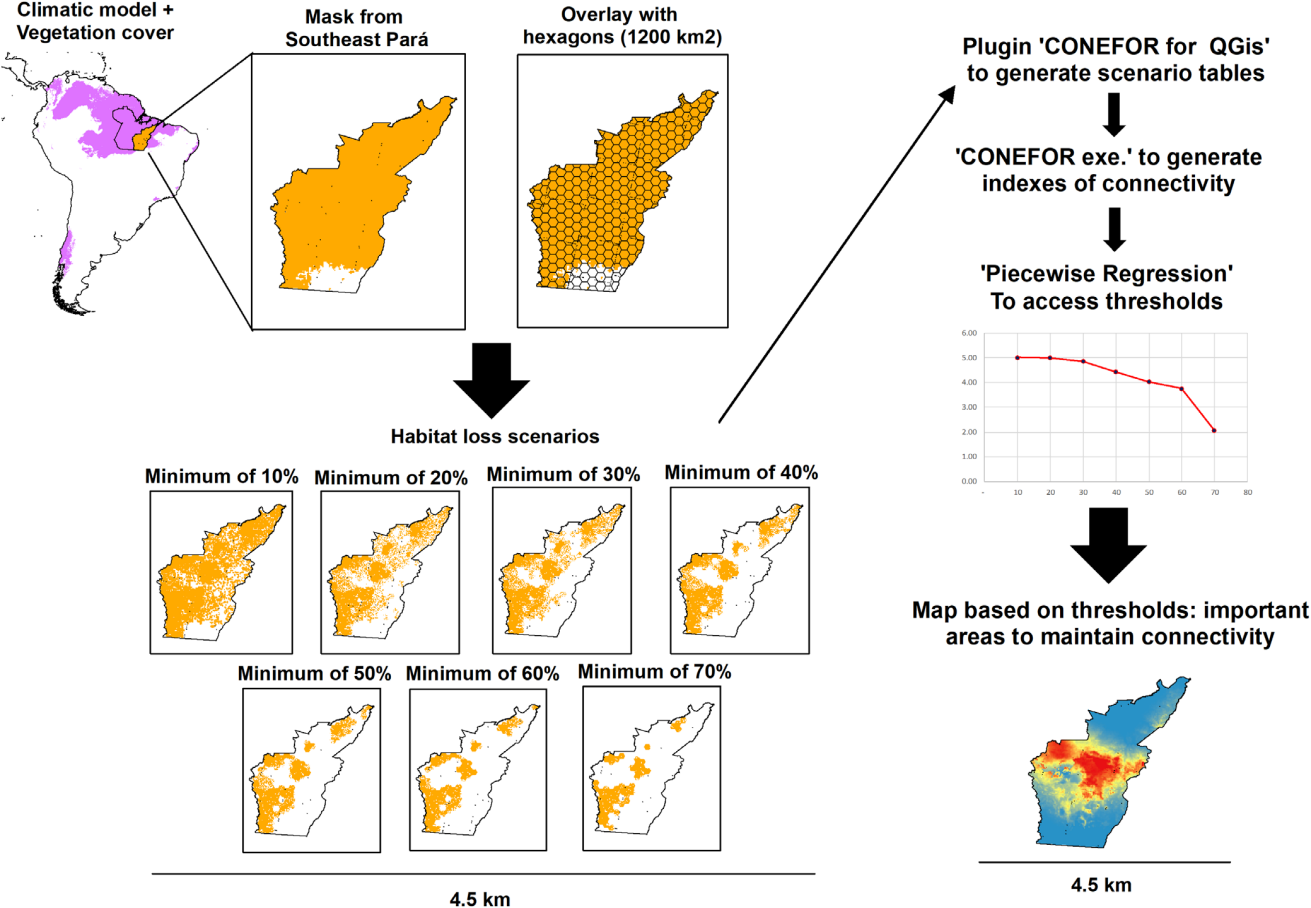
Flowchart of the main methodological steps of the study. Climatic models combined with the vegetation layer to define the available habitat for each species, which were overlaid with a grid of hexagons representing landscapes within the study area. We created scenarios retaining landscape with at least 10%, 20%, 30%, 40%, 50%, 60%, and 70% of the available habitat, which were converted into distance matrices between habitat patches to generate functional connectivity indices. Finally, a piecewise regression was performed on the index values to identify breakpoints that indicated the habitat amount scenario best representing the thresholds for each species. The selected scenarios were used to produce richness maps, highlighting priority areas to enhance functional connectivity for species.

This study was conducted in the Carajás highlands and surrounding areas within the southeastern mesoregion of Pará state in Brazil (Figure 1). The region exhibits high environmental heterogeneity and is characterized by a mosaic of natural and anthropogenic activity. It features residual highlands with ferruginous rocky outcrops and plateaus (up to 700 m) covered by unique savannah‐like vegetation known as *canga* vegetation (Devecchi et al. 2020), embedded within a matrix of original lowland Amazon rainforest that is now interspersed with pasture, agriculture, and urban land cover (Viana et al. 2016; Giulietti et al. 2019). This anthropogenic mosaic extends throughout the southeastern mesoregion, where adjacent protected areas, collectively known as the Carajás Mosaic (Federal Decree No. 2486), serve as refuges for regional biodiversity. These protected areas include the Carajás National Forest (Flona de Carajás), Tapirapé‐Aquiri National Forest (Flona Tapirapé‐Aquiri), Itacaiúnas National Forest, Tapirapé Biological Reserve (Rebio de Tapirapé), and the Igarapé Gelado Environmental Protection Area (APA do Igarapé Gelado).

### 2.2 Species and Occurrence Points

**FIGURE 2:**
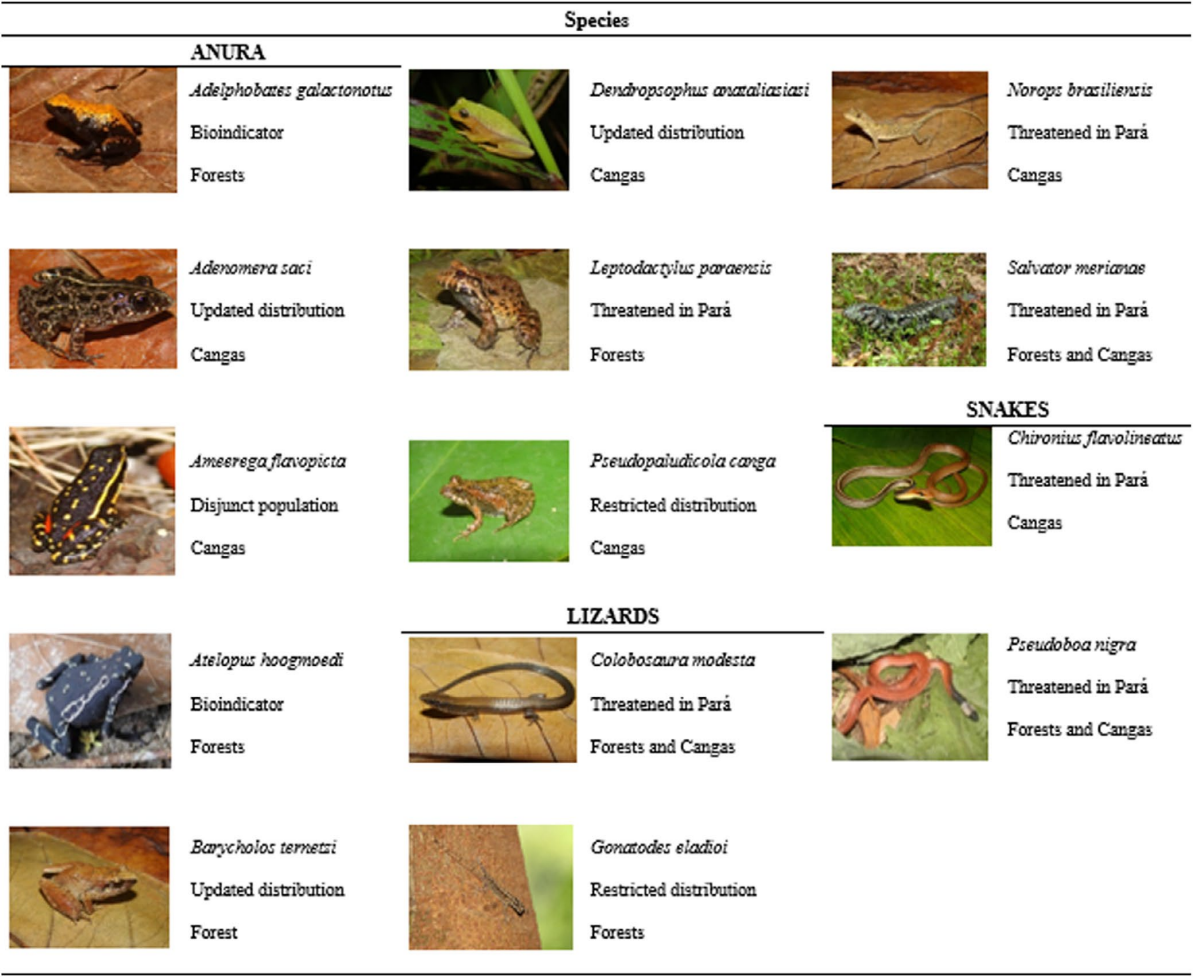
List of analyzed species with their respective conservation interest (in the state of Pará) and habitat. Photo credits: *Adelphobates galactonotus* , *Adenomera saci* , *Barycholos ternetzi* , *Dendropsophus anataliasiasi* , *Leptodactylus paraensis* , *Pseudopaludicola canga* , *Colobosaura modesta*, *Norops brasiliensis*, *Salvator merianae*, *and Chironius flavolineatus*: Fernando Rojas‐Hunjaic; *Ameerega flavopicta* , *Atelopus hoogmoedi* , *and Pseudoboa nigra*: Cássia Teixeira. *Gonatodes eladioi* : Sidnei Dantas.

For this study, we initially compiled a list of amphibians and Squamata reptile species from southeastern Pará, occurring in forest and canga ecosystems. We then filtered this list for endemic species considered threatened according to the list of species threatened with extinction in Pará (IDEFLOR‐Bio 2016), as well as species present in mining areas subject to environmental licensing processes that require impact quantification (ICMBio 2020). Based on these criteria, 14 species were selected, including eight anurans, four lizards, and two snakes (Figure 2). Occurrence records for these species were obtained from the following online databases: GBIF (http://www.gbif.org/, Table S1), SpeciesLink (http://splink.cria.org.br/), and SiBBr (https://sibbr.gov.br/). Additionally, we used occurrence data from specimens deposited in the Herpetological Collection of the Museu Paraense Emílio Goeldi (MPEG). Occurrence coordinates were reviewed, and we performed a cleaning to eliminate doubtful records, such as incomplete coordinates, state centroids, and coordinates outside the known distribution of the species (Table S1). Subsequently, records were overlapped within our study region and simplified to only one occurrence per pixel to reduce geographic sampling bias.

### 2.3 Species Distribution Models

Bioclimatic models must be generated across the entire species distribution to capture the full range of bioclimatic variability (Peterson 2003), even if the area of interest is only a subset of the distribution. Therefore, we considered the entire South American continent for generating bioclimatic models, as several species extend into other biomes beyond the Amazon. To avoid arbitrary selection of climate predictors, we used 19 present‐day bioclimatic variables from data collected from the 1970s to 2000s, and elevation due to the high altitude variation present in the study region. All variables were obtained from the WorldClim online platform (version 2.1) (Hijmans et al. 2005; https://www.worldclim.org/) with a resolution of 20.25 km^2^ (~4.5 km side = 2.5 arc‐minutes), also adopted for this study as it is considered reasonable for analyses of large geographic areas. We performed a principal component analysis (PCA), which reduced the data to fewer dimensions (principal components) without losing information about data variability. The first six axes explained 96% of the data variation, and we used them as predictor variables to minimize collinearity among highly correlated variables (Table S2).

SDMs were generated using three machine learning algorithms known for their efficiency in modeling species distribution (Elith et al. 2006; Meynard and Quinn 2007; Oppel et al. 2012): the “Maximum Entropy (MaxEnt),” a presence‐background algorithm based on the principle maximum entropy (Phillips et al. 2006); and “Random Forest,” which constructs multiple decision trees from randomly defined subsets of data and selects the most recurrent and probable distribution (Schapire 2001), and “Support Vector Machine (SVM),” a probability class‐based algorithm (Guo et al. 2005; Phillips et al. 2006), both presence/absence approaches. Given that the original dataset consists of presence‐only (occurrence) data, we generated an equal amount of pseudo‐absences by randomly sampling from the background within the study region. However, pseudo‐absence sampling was environmentally restricted to cells with lower suitability values (equal to or below 0.01), predicted by an exploratory Bioclim model (Andrade et al. 2020; Engler et al. 2004).

We employed the “checkerboard method” to partition the data into two spatially structured subsets, a training subset used to fit the models and another to validate the adjusted models, a common approach in SDM evaluation (Muscarella et al. 2014; Velazco et al. 2017). This technique reduces the influence of spatial autocorrelation between the training and testing data by dividing the study area into a checkerboard‐like grid of alternating patches and selecting data points from different patches. The patch size is based on the grid that maximizes spatial independence (Moran's *I*), environmental similarity, and the number of points in each subset to obtain the best representation and balance of occurrence points. Grids of different sizes were generated for each species, varying between 2 and 20 patches.

For every model run for each species, we derived thresholds from the “receiver operating characteristic” (ROC) curve, selecting the point that best balances errors of omission and commission, allowing the conversion of climate suitability predictions into presence/absence distribution maps (Phillips et al. 2006). To evaluate the performance of our generated models, we used two metrics: the “area under the curve” (AUC) and the “true skill statistic” (TSS). The AUC ranges from 0 to 1, with values ≤ 0.5 indicating models performing no better than random chance, and the TSS ranges from −1 to 1, with values close to or below 0 also representing performances no better than random. We considered values ≥ 0.7 for both metrics as our criteria for satisfactory performance (Allouche et al. 2006; Girardello et al. 2009). We generated three models for each species, one for each algorithm used. To obtain an ensemble model, we calculated the arithmetic mean of the models that met satisfactory performance criteria based on AUC and TSS. All procedures were conducted using the ENMTML package (Andrade et al. 2020), available on GitHub (https://github.com/andrefaa/ENMTML), within the R 2.1.0 programming environment (R Core Team 2022). The code used for this study is also available as a Supporting Information (Code S1).

### 2.4 Landscape Analysis

To proceed with landscape analyses, we first cropped the SDMs generated for South America to the extent of our study region in southeastern Pará. These cropped SDMs, representing each species' potential bioclimatic distribution in the region, were then used as masks to extract relevant cells from current land use and land cover maps, defining habitat availability. We used the 2021 MapBiomas project rasters (https://mapbiomas.org/; Souza et al. 2020) and applied nearest‐neighbor resampling to match their resolution to the cropped SDMs. To account for habitat preferences, we selected vegetation types relevant for each species: forest and savanna—the latter being the MapBiomas classification most compatible with canga areas in southeastern Pará. We then overlaid these vegetation layers with each species' high bioclimatic suitability cells to produce estimates of available habitat; this definition was standardized throughout the text. As a result, forest species were mapped on the forest layer, savanna species onto the savanna layer, and species occurring in both environments onto both layers.

Next, we overlaid our study region with a grid of hexagons, each representing a landscape sample with an area of 1200 km^2^, further dividing species' available habitat. The dimensions of the landscape samples used here were based on previous studies defining local landscapes for amphibian species (Becker et al. 2010). This procedure resulted in landscape samples varying from 1% to 100% of available habitat.

To assess how habitat availability in landscape samples influences regional connectivity for each species, we first calculated regional connectivity using the total available habitat. We then repeated the calculations under seven habitat amount scenarios, progressively excluding landscapes with less than 10%, 20%, 30%, 40%, 50%, 60%, and 70% habitat. For example, in the 60% scenario, only landscapes with at least 60% habitat were retained for regional connectivity calculations, while those below this threshold were removed. This iterative process was applied to each species based on the assumption that landscape samples with less habitat than the scenario's threshold could not maintain local connectivity, and we investigated how this impacted regional connectivity.

### 2.5 Connectivity Index

Functional connectivity can be measured by quantifying the importance of a patch and the functional connections with other landscape elements (Saura and Torné 2009). In this study, connectivity was evaluated in two steps: first, by calculating the connectivity metric for each species under each scenario, and second, by using these calculations to identify a habitat amount threshold below which landscape connectivity begins to erode.

Functional connectivity was assessed using the delta integral connectivity index (dIIC) and its three components: dIICintra, dIICflux, and dIICconnector. The integral connectivity index (IIC) is a binary index that evaluates the presence or absence of connectivity between two habitat fragments, allowing for the assessment of a fragment's role in overall landscape connectivity or connectivity among fragment combinations (Pascual‐Hortal and Saura 2006; Saura and Torné 2009). Based on graph theory, the index represents landscape heterogeneity as a network of nodes (representing patches) connected by lines (inter‐patch connections) (Bunn et al. 2000; Urban and Keitt 2001; Minor and Urban 2008). Essentially, the IIC measures the available habitat within the landscape (Pascual‐Hortal and Saura 2006; Saura and Pascual‐Hortal 2007).

The IIC ranges from 0 to 1, with higher values indicating greater connectivity. IIC is calculated using the following formula: $\mathrm{IIC}=\frac{\sum_{i=1}^{n}\sum_{j=1}^{n}\frac{a_{i}xa_{j}}{1+{\mathrm{nl}}_{\mathrm{ij}}}}{A_{L}^{2}}$, where *n* is the total number of nodes in the landscape, *a*_*i*_ is the area of each fragment, *𝑛𝑙*_*ij*_ is the number of links in the least‐cost path between fragments *i* and *j*, and *A*_L_ is the total landscape area, including both areas with and without fragments (Saura and Torné 2012). This formula integrates the spatial arrangement and size of habitat patches to evaluate overall connectivity in the landscape.

The dIIC quantifies the relative change in the IIC value of general connectivity across the entire landscape following the loss of a specific node (Saura and Rubio 2010; Saura and Torné 2012). The dIIC is calculated as follows: $\text{dIIC}\left(\%\right)=\frac{\mathrm{IIC}-{\mathrm{IIC}}^{i}}{\mathrm{IIC}}\times 100$, where IIC corresponds to the global index value calculated for the landscape considering all patches, and IIC^*i*^ is the global index value after removing fragment *i* from the landscape (Saura and Torné 2012; Arimoro 2015). The dIIC values are derived from three components based on how nodes or links contribute to connectivity: dIIC = dIICintra + dIICflux + dIICconnector. The dIICintra measures the contribution of connectivity within the fragment to overall landscape connectivity, independent of patch connections, dispersal distances, and isolation in the landscape. The dIICflux reflects the degree to which a patch is connected to others but does not evaluate its significance to the landscape. The dIICconnector assesses the role of a fragment or link in contributing to connectivity between other fragments, functioning as a connection or stepping stone (Saura and Rubio 2010). We set the dispersal distance at 1 km, which is the maximum value at which two nodes can be considered connected. Above this value, connections are disregarded.

The known dispersal distances for amphibians can range from 200 to 2000 m (Smith and Green 2005; Kovar et al. 2009). However, data on tropical species for both amphibians and reptiles is scarce. In the absence of consistent information on the movement of amphibians and reptiles, we performed an initial sensitivity analysis using dispersal distance values of 500, 1000, and 1500 m (values within the mentioned literature data) and found only minor variation between them in calculated IIC values (Table S6). Thus, we used the 1000 m value on further analyses, representing the dispersal capacity of a generic species with limited dispersal capacity (Diniz et al. 2018). All IIC values were calculated using the “CONEFOR for Qgis” plugin, these rasters were converted into distance matrices between habitat patches, which were later entered into the “CONEFOR.exe” application to generate functional connectivity indices.

### 2.6 Connectivity Threshold

We investigated thresholds in the amount of habitat within landscapes beyond which regional connectivity is disrupted for species in southeastern Pará. Using piecewise regression, we modeled the IIC values calculated as a function of variable minimum amounts of habitat in the landscape (10%, 20%, 30%, 40%, 50%, 60%, and 70%). This approach consists of a segmented linear regression function, where the models include two or more lines connected by “breakpoints,” which serve as threshold estimates (Toms and Lesperance 2003). We expect to find a better fit for the data with segmented regression, where breaks in the modeled trends indicate the threshold we are evaluating. These procedures were conducted in the R environment (R Core Team 2022), utilizing the “ggplot2” packages (Wickham 2016) for visualization and the “segmented” package (Muggeo 2003; Muggeo and Muggeo 2017) for regression analysis, and the code is available as a Supporting Information (Code S2).

We used the threshold identified by the piecewise regression to define the most critical landscapes for maintaining connectivity for all analyzed species. Finally, we added up these landscapes in a GIS environment to create a final map illustrating the overlap of these critical areas, separately for forest and savanna species (Figure 1).

## 3 Results

### 3.1 Species Distribution Models

Among the analyzed species, *Salvator merianae* Duméril & Bibron, 1839 had the highest number of unique occurrence points (*N* = 211), while *Adenomera saci* Carvalho & Giaretta, 2013 had the fewest (*N* = 9; Table S3). None of the species was considered globally threatened (IUCN 2020) or nationally threatened (MMA 2022). However, six species were listed as regionally threatened in Pará (IDEFLOR‐Bio 2016) (Figure 2). All models demonstrated excellent predictive capacity, with AUC values ranging from 0.99 to 1 and TSS values ranging from 0.99 to 1 (Table S3).

### 3.2 Landscape Analysis

The largest habitat area available for a species in our study region, measured by the number of pixels, was 7363 (equivalent to 149,100 km^2^) for *Colobosaura modesta* (Reinhardt & Lütken, 1862), while the smallest was 113 (equivalent to 2288 km^2^) for *Dendropsophus anataliasiasi* (Bokermann, 1972) (Table S4). The largest habitat areas were observed for forest‐associated species, including both strictly forest‐dwelling taxa and those that also occur in savannas. In contrast, savanna‐exclusive species exhibited highly fragmented and spatially dispersed habitat areas, primarily concentrated in the southern portion of the study region (Figure S1). The Carajás Mosaic of PA encompassed the greatest extent of suitable habitat for forest species, underscoring its importance as a key refuge for regional biodiversity.

Similarly, the overall habitat availability for forest‐associated species was relatively well distributed across all habitat amount scenarios, indicating that these species occur in landscapes ranging from near‐intact to relictual conditions within the study region. This pattern is illustrated by *Adelphobates galactonotus* (Steindachner, 1864), as shown in Figure S2. In contrast, the savanna‐exclusive species *Adenomera saci* , *Ameerega flavopicta* (Lutz, 1925), *Dendropsophus anataliasiasi* , *Norops brasiliensis* (Vanzolini & Williams, 1970), *Chironius flavolineatus* (Jan, 1863), and *Pseudopaludicola canga* Giaretta & Kokubum, 2003, were absent from landscapes containing 70%, 60%, 50%, and 40% habitat cover. Moreover, no landscapes with 30% habitat cover were recorded for *Pseudopaludicola canga* (Table S4).

### 3.3 Connectivity Index

**FIGURE 3:**
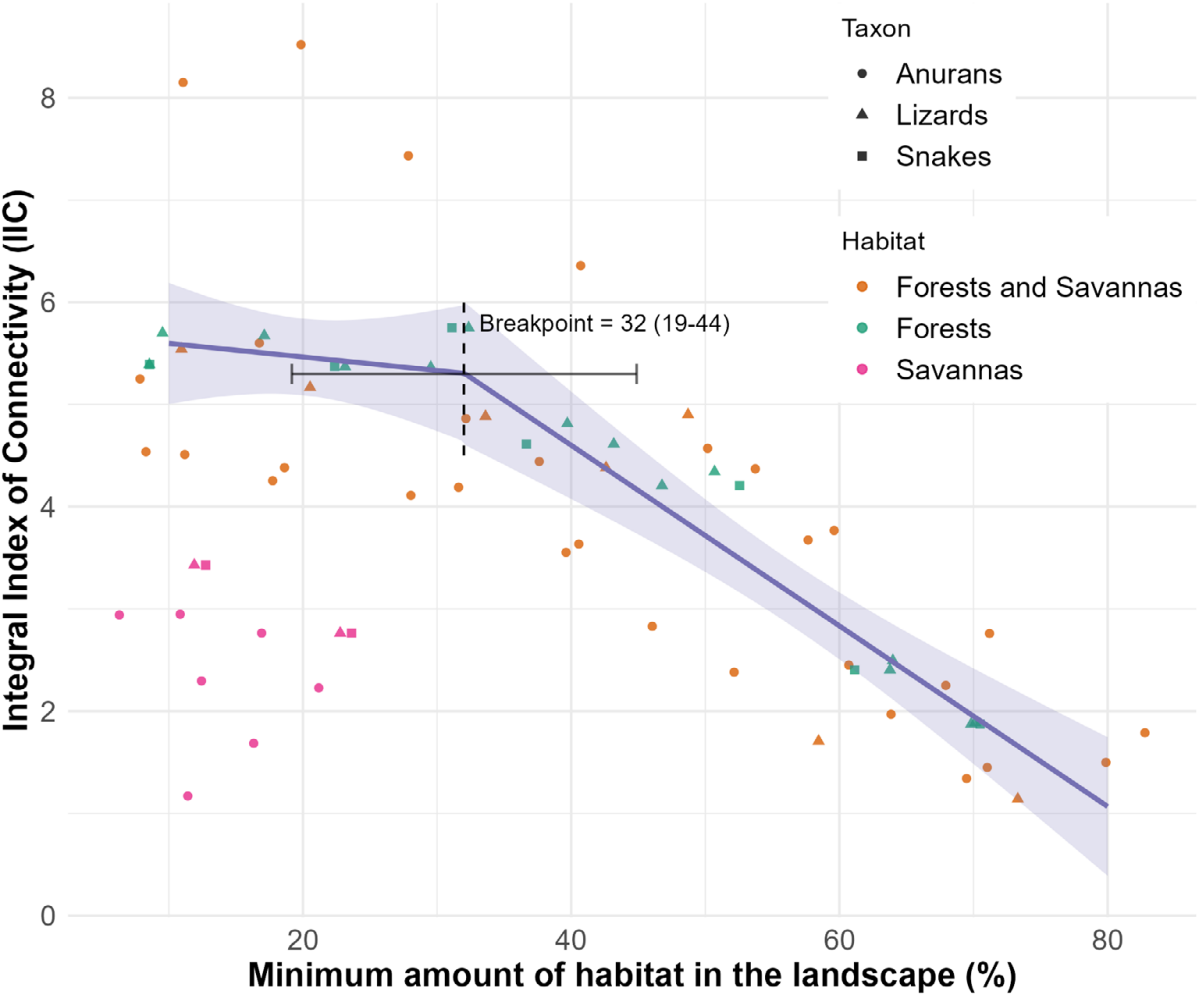
Segmented regression exploring thresholds of habitat amount in the landscape for the herpetofauna of Southeastern Amazonia. Data points show integral connectivity index (IIC) values for different habitat quantity scenarios in the landscape, depicting different habitats and taxa of the herpetofauna in Southeast Pará. The piecewise regression was conducted considering only forest‐exclusive species and forest and savanna species, indicating a critical threshold of 32% of habitat cover (95% CI: 19.2%–44.9%) in habitat amount, disrupting the relationship with regional connectivity.

The IIC values were the highest when landscape samples with a minimum of 10% of habitat were maintained in the scenario and decreased as the minimum habitat threshold increased (Table S5). This indicated a consistent pattern across all species suggesting that low habitat amount landscapes are contributing to maintaining overall connectivity in the study region. A notable difference was related to the extent of habitat available for forest species (including forest and savanna species), compared to canga exclusive species (Figure 3). For savanna species, only the scenarios with 10% and 20% habitat quantities were used for IIC values, as scenarios with 30% habitat did not provide reliable results, deviating from the observed pattern for the forested species.

### 3.4 Connectivity Threshold

We performed a piecewise regression exclusively for forest‐associated species (exclusively or forest‐savanna), revealing a significant breakpoint in habitat connectivity at 32% habitat cover (95% CI: 19.2%–44.9%; *p* = 0.026; *R* ^2^ = 0.71; Figure 3). The segmented model had a lower AIC (ΔAIC = −4.13) compared to the linear model, indicating improved explanatory power. Savanna‐exclusive species, whose landscape samples had below 30% habitat availability for all species and IIC values were consistently smaller, were not modeled (Figure 3).

### 3.5 Priority Areas for Conservation

Considering only forest species (including forest and savanna species), the results suggest that landscapes with at least 30% habitat quantity are critical for maintaining overall connectivity. For the savanna species, we considered all available habitat areas critical since they have at most 20% habitat quantity in the landscape. For each species, we identified the importance value of each node (patch) in the evaluated area using the dIIC metric (Figure S3). For forest species, observing larger areas of critical patches for connectivity is possible. In contrast, these critical areas are strongly reduced to fragmented points for savanna species, primarily concentrated in the southeastern part of the study area.

**FIGURE 4:**
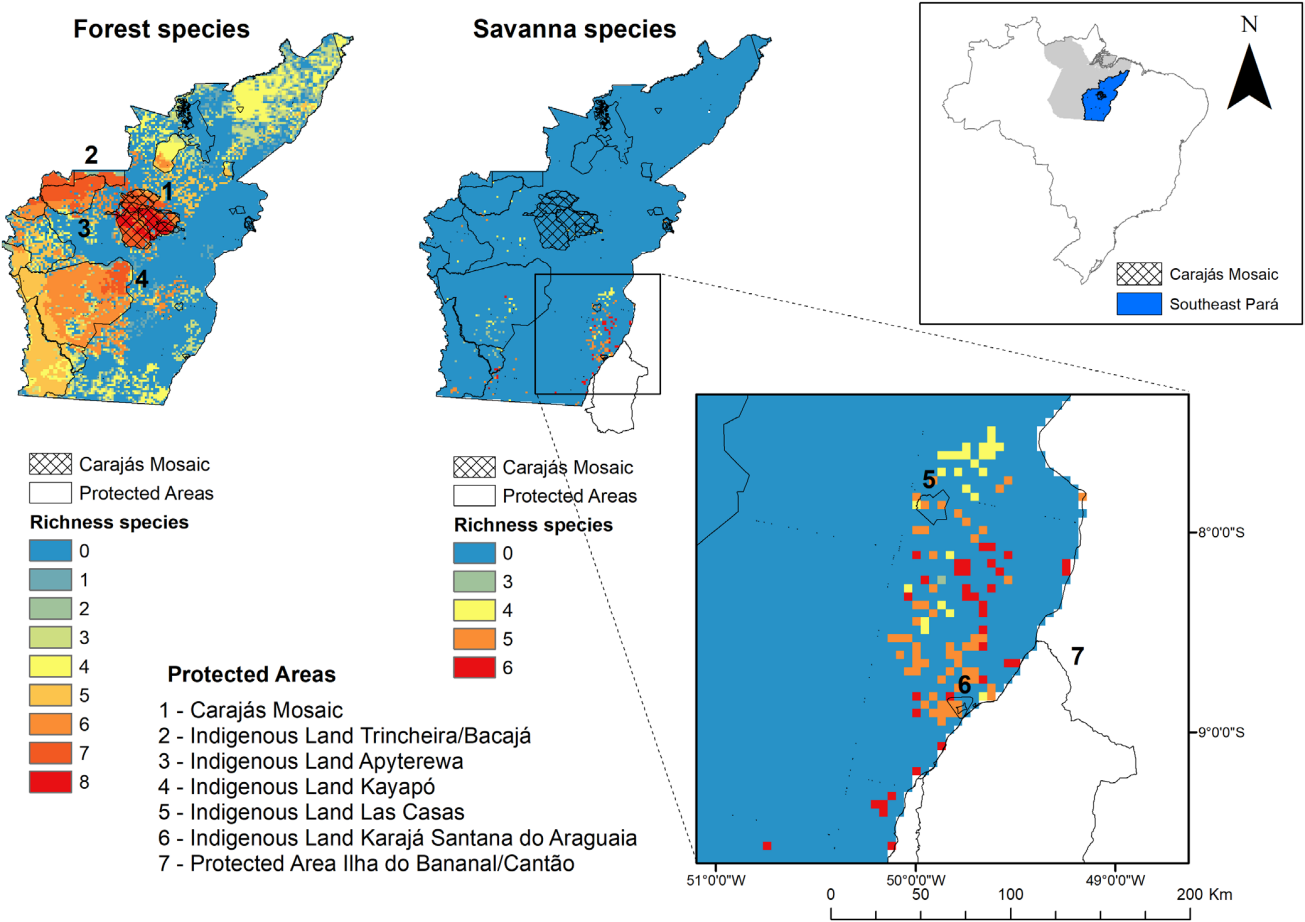
Location of the study area in the southeast of the State of Pará, highlighting the Carajás Mosaic region. The areas are identified as most important for maintaining full landscape connectivity for both forest species (including forest and savanna species) and *savanna* species in southeast Pará.

Regarding the conservation status of these species and the extent of habitat areas within protected areas, forest species accounted for about 59% of their areas within conservation units. In contrast, the savanna species had only 20% (Figure 4). This indicates that savanna species have few habitat areas, and a low proportion of these areas are protected. For conservation planning, we overlaid the most important areas for each species. We found that for forest species, the Carajás Mosaic is crucial as a common area for all of them, followed by the Kayapó and Apyterewa indigenous lands. In contrast, the most important areas for savanna species are small fragmented areas in the southeastern part of the study area, all outside conservation units (Figure 4).

## 4 Discussion

ETH proposes that below a critical threshold of habitat in a landscape, landscape connectivity erodes, and species persistence becomes dependent on the size and isolation of remaining patches (Metzger and Décamps 1997; Fahrig 2003), which has been influential in linking landscape context with local patch‐area effects (Pardini et al. 2010). Our study builds on this framework by extending it to regional connectivity. We demonstrated that if the herpetofauna species cannot persist in landscapes with less than ~32% habitat cover (or 44% if we want to be conservative and aim to the upper 95% CI), the overall regional connectivity in Southeastern Amazonia is expected to collapse. Our results in the present study aimed to further link the availability of areas of high environmental suitability and the effects of landscape changes.

As expected, the forest species analyzed had a more extensive habitat than the *canga* species, which had more restricted and isolated habitat areas. Our results indicate that maintaining a forest habitat quantity of approximately 32% of a landscape is necessary to prevent a significant decline in regional connectivity. In contrast, *canga* species were not tested in this context, as their landscapes are already well below this theoretical threshold. *Cangas* are naturally restricted, covering about 115 km^2^ in southeastern Pará, with most of this formation occurring within the Carajás National Forest (FLONA de Carajás) and the National Park of Ferruginous Fields (PNCF) (Giulietti et al. 2019). Therefore, we can assume that a critical habitat threshold works well for forest species that originally had extensive habitat areas.

Regardless of anthropogenic alterations, Savanna enclaves in the Amazon are naturally small and fragmented, representing mainly “shrub and grassland islands” amid the forest (Martins et al. 2012). Consequently, the species evaluated in this environment, which only presented landscape samples with up to 25% habitat quantity, were constrained by the geographical restrictions of the savannas. Thus, our main prediction that *canga* species have less connectivity and are more vulnerable compared to forest species was supported. The observed low connectivity in *cangas* may be isolating the FLONA de Carajás and PNCF species. For amphibians, species are expected to decline even within protected areas due to climate change (Lemes et al. 2014). Moreover, industrial activities such as mining, are allowed in the FLONA de Carajás. Therefore, it is a strategic to also focus in *canga* areas outside the protected areas in southeastern Pará, focusing in preserving or restoring these canga patches.

The regional functional connectivity of species was higher when landscapes with at least 10% of habitat were included. While many landscape samples contained low amounts of habitat, despite their perceived isolation, habitat patches within these landscapes were sufficient to contribute to regional connectivity. ETH proposes that low‐habitat landscapes transition from a well‐connected system to one dominated by small, isolated patches (Swift and Hannon 2010). Small patches offer fewer resources for species with higher interior habitat demands and low mobility, often jeopardizing their persistence (Laurance 1990). On the other hand, increasing evidence also points to small fragments as crucial connections between larger patches, thereby reducing overall landscape connectivity resistance (Luck and Daily 2003; Ribeiro et al. 2009; Mueller et al. 2014; de Oliveira‐Junior et al. 2020). Furthermore, a set of small fragments may be less susceptible to stochastic effects than a single isolated fragment, reducing the risk of metapopulation extinction (Riva and Fahrig 2022). Low‐habitat anthropogenic landscapes are becoming more common in eastern Amazonia; therefore, small fragments may play an important role in biodiversity conservation than previously expected (Shafer 1995).

In this study, we used the same scale and resolution for all species in the analytical steps to facilitate the comparison of connectivity indices and to identify valid priority areas for most of them. However, amphibians and squamate reptiles have ecological requirements beyond habitat quantity alone. Amphibians, for instance, depend on humid environments to complete their aquatic life stages; thus, the presence and connectivity of terrestrial environments and water bodies are essential for breeding site availability and maintaining functional diversity (Dayrell et al. 2024). Therefore, even with *cangas*, species‐specific needs must be included in the results and conservation planning discussions, especially for low‐habitat landscapes.

For forest species, the Carajás Mosaic and IRs located in southeastern Pará constitute a large complex of areas with the potential to be connected by ecological corridors. The results point to the role of protected areas as essential barriers to biodiversity loss (UNEP‐WCMC and IUCN 2023; Begotti and Peres 2020), as forest areas outside these units are virtually nonexistent. Therefore, investment in combating illegal activities within these areas is extremely necessary. A study in the Xingu Endemism Area pointed out that PAs, IRs, and forest fragments in the western portion have a high potential for connectivity (Castro et al. 2020). In our study, we observed that for *canga* species, the most important areas are located outside protected areas, specifically within the municipalities of Santana do Araguaia, Pau D'Arco, Redenção, Conceição do Araguaia, Floresta do Araguaia, and Santa Maria das Barreiras, highlighting the risk of local extinction for these species. These municipalities are located further south in Pará, near the border with the states of Maranhão and Tocantins. This region is characterized by high deforestation rates associated with agricultural expansion and land conflicts. Major economic activities include agriculture, logging, and mining (Homma et al. 2014). Conservation policies for these species should focus on these municipalities, considering the intense history of environmental change and the limited extent of the natural *canga* remnants.

The information generated in this study indicates that conservation policies integrating functional connectivity must consider that, for forest species, a minimum habitat threshold of 35% is necessary to maintain connectivity. Since the areas important for the conservation of these species are almost entirely within conservation units, they are of great importance for their preservation, particularly Indigenous lands. These have shown significant importance for the functional connectivity of forest species, primarily due to their larger geographic extents. Specifically, the anuran species *Atelopus hoogmoedi* Lescure is identified as a bioindicator, and the drastic decline observed in various species within the genus indicates a high level of vulnerability (Luger et al. 2009; Lötters et al. 2023), which has not yet been formally assessed in any risk evaluation process. For this species, the Carajás Mosaic and, especially, the Trincheira/Bacajá Indigenous Territory have been identified as important for functional connectivity. However, these areas are not strongly connected to each other, suggesting a potential conservation interest area.

For this reason, forest species can be considered less vulnerable. In contrast, canga species have areas of disconnected habitat outside protected areas, making them critical targets for conservation planning. Brazil has established the National Plan for the Recovery of Native Vegetation (Planaveg), which promotes public policies to encourage vegetation recovery and mobilize financial resources for restoration. Given our results, we believe restoration strategies integrated into Planaveg should be aimed at restoring low‐habitat landscapes which were important for enhancing regional connectivity. As in the southeastern region of Pará, the warm‐colored fragments in Figure 4 may represent areas of interest for restoration and, consequently, increase the connection to the Kayapó Indigenous land, a large and more continuous fragment.

## Author Contributions

**Cássia Teixeira:** conceptualization (equal), data curation (lead), formal analysis (lead), investigation (equal), methodology (equal), writing – original draft (lead), writing – review and editing (lead). **Gisele Lopes Nunes:** data curation (supporting), funding acquisition (lead), investigation (supporting), resources (equal), writing – review and editing (equal). **Leonardo Carreira Trevelin:** conceptualization (equal), formal analysis (supporting), investigation (equal), methodology (equal), validation (equal), writing – review and editing (equal). **Daniel Paiva Silva:** formal analysis (supporting), methodology (supporting), supervision (supporting), validation (equal), writing – review and editing (equal). **Ana Lúcia da Costa Prudente:** conceptualization (supporting), investigation (equal), methodology (supporting), project administration (lead), resources (equal), supervision (lead), validation (equal), writing – review and editing (equal).

## Conflicts of Interest

G.L.N. and L.C.T. work for a non‐profit research institution funded by the private mining company Vale S.A. However, this organization had no role in the study design, data collection, formal analysis, interpretation of results, manuscript preparation, or the decision to publish the findings. All authors declare no other potential conflicts of interest.

## Supporting information


Data S1.

Data S2.

## Data Availability

The authors confirm that the data and codes supporting the results of this study are available in the Supporting Information or can be accessed on GitHub at: https://github.com/andrefaa/ENMTML. Climatic data were downloaded from https://www.worldclim.org/ and land cover maps were downloaded from https://mapbiomas.org.

## References

[ece371741-bib-0001] Allouche, O. , A. Tsoar , and R. Kadmon . 2006. “Assessing the Accuracy of Species Distribution Models: Prevalence, Kappa and the True Skill Statistic (TSS).” Journal of Applied Ecology 43: 1223–1232. 10.1111/j.1365–2664.2006.01214.x.

[ece371741-bib-0002] Andrade, A. F. A. , S. J. E. Velazco , and P. De Marco Júnior . 2020. “ENMTML: An R Package for a Straightforward Construction of Complex Ecological Niche Models.” Environmental Modelling & Software 125: 104615.

[ece371741-bib-0003] Andrén, H. 1994. “Effects of Habitat Fragmentation on Birds and Mammals in Landscapes With Different Proportions of Suitable Habitat: A Review.” Oikos 71: 355–366.

[ece371741-bib-0004] Arimoro, O. A. S. 2015. “Uso de geotecnologias aplicadas em estudos de modelos de ocupação e conectividade para mamíferos de médio e maior porte no Cerrado.” Masters' diss., Universidade de Brasília, Instituto de Geociências.

[ece371741-bib-0005] Arroyo‐Rodriguez, V. , L. Fahrig , M. Tabarelli , et al. 2020. “Designing Optimal Human‐Modified Landscapes for Forest Biodiversity Conservation.” Ecology Letters 23: 1404–1420. 32537896 10.1111/ele.13535

[ece371741-bib-0006] Barlow, J. , G. D. Lennox , J. Ferreira , et al. 2016. “Anthropogenic Disturbance in Tropical Forests Can Double Biodiversity Loss From Deforestation.” Nature 535: 144–147. 27362236 10.1038/nature18326

[ece371741-bib-0007] Becker, C. G. , R. D. Loyola , C. F. B. Haddad , and K. R. Zamudio . 2010. “Integrating Species Life‐History Traits and Patterns of Deforestation in Amphibian Conservation Planning.” Diversity and Distributions 16: 10–19.

[ece371741-bib-0008] Begotti, R. A. , and C. A. Peres . 2020. “Rapidly Escalating Threats to the Biodiversity and Ethnocultural Capital of Brazilian Indigenous Lands.” Land Use Policy 96: 1–10.

[ece371741-bib-0009] Bunn, A. G. , D. L. Urban , and T. H. Keitt . 2000. “Landscape Connectivity: A Conservation Application of Graph Theory.” Journal of Environmental Management 59: 265–278.

[ece371741-bib-0010] Castro, R. B. , J. L. G. Pereira , R. Saturnino , P. S. D. Monteiro , and A. L. K. M. Albernaz . 2020. “Identification of Priority Areas for Landscape Connectivity Maintenance in the Xingu Area of Endemism in Brazilian Amazonia.” Acta Amazonica 50: 68–79.

[ece371741-bib-0011] Cowling, S. A. , R. A. Betts , P. M. Cox , et al. 2004. “Contrasting Simulated Past and Future Responses of the Amazonian Forest to Atmospheric Change.” Philosophical Transactions of the Royal Society of London. Series B, Biological Sciences 359: 539–547. 15212101 10.1098/rstb.2003.1427 PMC1693338

[ece371741-bib-0012] Dayrell, S. S. , R. Fraga , C. A. Peres , P. E. D. Bobrowiec , and W. E. Magnusson . 2024. “Functional Responses of Amazonian Frogs to Flooding by a Large Hydroelectric Dam.” Biodiversity and Conservation 33: 1–16.

[ece371741-bib-0013] de Oliveira‐Junior, N. D. , G. Heringer , M. L. Bueno , V. Pontara , and J. A. A. Meira‐Net . 2020. “Prioritizing Landscape Connectivity of a Tropical Forest Biodiversity Hotspot in Global Change Scenario.” Forest Ecology and Management 472: 118247.

[ece371741-bib-0014] Devecchi, M. F. , J. Lovo , M. F. Moro , et al. 2020. “Beyond Forests in the Amazon: Biogeography and Floristic Relationships of the Amazonian Savannas.” Botanical Journal of the Linnean Society 193, no. 4: 478–503.

[ece371741-bib-0015] Diniz, M. F. , R. B. Machado , A. A. Bispo , and P. De Marco Júnior . 2018. “Can We Face Different Types of Storms Under the Same Umbrella? Efficiency and Consistency of Connectivity Umbrellas Across Different Patchy Landscape Patterns.” Landscape Ecology 33: 1911–1923.

[ece371741-bib-0016] Dobrovolski, R. , R. Loyola , L. Rattis , et al. 2018. “Science and Democracy Must Orientate Brazil's Path to Sustainability.” Perspectives in Ecology and Conservation 16: 121–124.

[ece371741-bib-0017] Elith, J. , C. H. Graham , R. P. Anderson , et al. 2006. “Novel Methods Improve Prediction of Species' Distributions From Occurrence Data.” Ecography 29: 129–151.

[ece371741-bib-0018] Engler, R. , A. Guisan , and L. Rechsteiner . 2004. “An Improved Approach for Predicting the Distribution of Rare and Endangered Species From Occurrence and Pseudo‐Absence Data.” Journal of Applied Ecology 41, no. 2: 263–274. 10.1111/j.0021–8901.2004.00881.x.

[ece371741-bib-0019] Fahrig, L. 1997. “Effects of Habitat Loss and Fragmentation on Population Dynamics.” Journal of Wildlife Management 61: 603–610.

[ece371741-bib-0020] Fahrig, L. 2003. “Effects of Habitat Fragmentation on Biodiversity.” Annual Review of Ecology, Evolution, and Systematics 34, no. 1: 487–515.

[ece371741-bib-0021] Fahrig, L. 2013. “Rethinking Patch Size and Isolation Effects: The Habitat Amount Hypothesis.” Journal of Biogeography 40: 1649–1663.

[ece371741-bib-0022] Fearnside, P. M. 2005. “Deforestation in Brazilian Amazonia: History, Rates, and Consequences.” Conservation Biology 19: 680–688.

[ece371741-bib-0023] Feeley, K. J. , and M. R. Silman . 2016. “Disappearing Climates Will Limit the Efficacy of Amazonian Protected Areas.” Diversity and Distributions 22: 1081–1084.

[ece371741-bib-0024] Fischer, J. , and D. B. Lindenmaye . 2007. “Landscape Modification and Habitat Fragmentation: A Synthesis.” Global Ecology and Biogeography 16: 265–280.

[ece371741-bib-0025] Girardello, M. , M. Griggio , M. J. Whittingham , and S. P. Rushton . 2009. “Identifying Important Areas for Butterfly Conservation in Italy.” Animal Conservation 12: 20–28.

[ece371741-bib-0026] Giulietti, A. M. , T. C. Giannini , N. F. Mota , et al. 2019. “Edaphic Endemism in the Amazon: Vascular Plants of the Canga of Carajás, Brazil.” Botanical Review 85: 357–383.

[ece371741-bib-0027] Gomes, V. H. F. , I. C. G. Vieira , R. P. Salomão , and H. ter Steege . 2019. “Amazonian Tree Species Threatened by Deforestation and Climate Change.” Nature Climate Change 9: 547–553.

[ece371741-bib-0028] Guo, Q. , M. Kelly , and C. H. Graham . 2005. “Support Vector Machines for Predicting Distribution of Sudden Oak Death in California.” Ecological Modelling 182: 75–90.

[ece371741-bib-0029] Haddad, N. M. , L. A. Brudvig , J. Clobert , et al. 2015. “Habitat Fragmentation and Its Lasting Impact on Earth's Ecosystems.” Science Advances 1: 1500052. 10.1126/sciadv.1500052 PMC4643828 26601154

[ece371741-bib-0030] Hijmans, R. J. , S. E. Cameron , J. L. Parra , P. G. Jones , and A. Jarvis . 2005. “Very High Resolution Interpolated Climate Surfaces for Global Land Areas.” International Journal of Climatology 25: 1965–1978.

[ece371741-bib-0031] Homma, A. K. O. , R. T. Walker , R. D. A. Carvalho , A. J. de Conto , and C. A. P. Ferreira . 2014. Políticas agrícolas e econômicas para a conservação de recursos naturais: o caso de castanhais em lotes de colonos no sul do Pará. Em: Extrativismo Vegetal na Amazônia: história, ecologia, economia e domesticação. EMBRAPA.

[ece371741-bib-0032] IDEFLOR‐Bio . 2016. O futuro da fauna ameaçada do Pará: Implicações para conservação da biodiversidade em diferentes cenários. Instituto de Desenvolvimento Florestal e da Biodiversidade.

[ece371741-bib-0033] IMAZON . 2023. Sistema de Alerta de Desmatamento Maio de 2021. Instituto do Homem e Meio Ambiente da Amazônia.

[ece371741-bib-0034] Instituto Chico Mendes de Conservação da Biodiversidade (ICMBio) . 2020. Ofício SEI n° 241/2020‐ICMBio Carajás. Instituto Chico Mendes de Conservação da Biodiversidade (ICMBio).

[ece371741-bib-0035] IUCN . 2020. The IUCN Red List of Threatened Species. International Union for Conservation of Nature.

[ece371741-bib-0036] Jaureguiberry, P. , N. Titeux , M. Wiemers , et al. 2022. “The Direct Drivers of Recent Global Anthropogenic Biodiversity Loss.” Science Advances 8, no. 45: eabm9982. 36351024 10.1126/sciadv.abm9982 PMC9645725

[ece371741-bib-0086] Kovar, R. , M. Brabec , R. Vita , and R. Bocek . 2009. “Spring Migration Distances of Some Central European Amphibian Species.” Amphibia‐Reptilia 30, no. 3: 367–378.

[ece371741-bib-0037] Laurance, W. F. 1990. “Comparative Responses of Five Arboreal Marsupials to Tropical Forest Fragmentation.” Journal of Mammalogy 71: 641–653.

[ece371741-bib-0038] Laurance, W. F. , D. Carolina Useche , J. Rendeiro , et al. 2012. “Averting Biodiversity Collapse in Tropical Forest Protected Areas.” Nature 489: 290–294. 22832582 10.1038/nature11318

[ece371741-bib-0039] Lemes, P. , A. S. Melo , and R. D. Loyola . 2014. “Climate Change Threatens Protected Areas of the Atlantic Forest.” Biodiversity and Conservation 23: 357–368.

[ece371741-bib-0040] Lötters, S. , A. Plewnia , A. Catenazzi , et al. 2023. “Ongoing Harlequin Toad Declines Suggest the Amphibian Extinction Crisis Is Still an Emergency.” Communications Earth & Environment 4: 412.

[ece371741-bib-0041] Lovejoy, T. E. , and C. A. Nobre . 2018. “Amazon Tipping Point.” Science Advances 4: 2340. 10.1126/sciadv.aat2340 PMC5821491 29492460

[ece371741-bib-0042] Luck, G. W. 2005. “An Introduction to Ecological Thresholds.” Biological Conservation 124, no. 3: 299–300.

[ece371741-bib-0043] Luck, G. W. , and G. C. Daily . 2003. “Tropical Countryside Bird Assemblages: Richness, Composition, and Foraging Differ by Landscape Context.” Ecological Applications 13: 235–247.

[ece371741-bib-0044] Luger, M. , W. Hödl , and S. Lötters . 2009. “Site Fidelity, Home Range Behaviour and Habitat Utilization of Male Harlequin Toads (Amphibia: *Atelopus hoogmoedi*) From Suriname: Relevant Aspects for Conservation Breeding.” Salamandra 45, no. 4: 211–218.

[ece371741-bib-0045] Marengo, J. A. , C. M. Souza , K. Thonicke , et al. 2018. “Changes in Climate and Land Use Over the Amazon Region: Current and Future Variability and Trends.” Frontiers in Earth Science 6: 1.

[ece371741-bib-0046] Martins, F. D. , A. F. Castilho , J. Campos , F. M. Hatano , and S. G. Rolim . 2012. Fauna da Floresta Nacional de Carajás: estudos sobre vertebrados terrestres. Nitro Editorial.

[ece371741-bib-0047] Metzger, J. P. , and H. Décamps . 1997. “The Structural Connectivity Threshold: An Hypothesis in Conservation Biology at the Landscape Scale.” Acta Oecologica 18, no. 1: 1–12.

[ece371741-bib-0048] Meynard, C. N. , and J. F. Quinn . 2007. “Predicting Species Distributions: A Critical Comparison of the Most Common Statistical Models Using Artificial Species.” Journal of Biogeography 34: 1455–1469.

[ece371741-bib-0049] Ministério do Meio Ambiente (MMA) . 2022. Portaria MMA No 148, de 7 de junho de 2022. Ministério do Meio Ambiente. Diário Oficial da União.

[ece371741-bib-0050] Minor, E. S. , and D. L. Urban . 2008. “A Graph‐Theory Framework for Evaluating Landscape Connectivity and Conservation Planning.” Conservation Biology 22: 297–307. 18241238 10.1111/j.1523-1739.2007.00871.x

[ece371741-bib-0051] Mueller, T. , J. Lenz , T. Caprano , W. Fiedler , and K. Böhning‐Gaese . 2014. “Large Frugivorous Birds Facilitate Functional Connectivity of Fragmented Landscapes.” Journal of Applied Ecology 51: 684–692.

[ece371741-bib-0052] Muggeo, V. M. 2003. “Estimating Regression Models With Unknown Break‐Points.” Statistics in Medicine 22, no. 19: 3055–3071. 12973787 10.1002/sim.1545

[ece371741-bib-0053] Muggeo, V. M. , and M. V. M. Muggeo . 2017. “Package ‘Segmented’.” Biometrika 58, no. 525–534: 516.

[ece371741-bib-0054] Muscarella, R. , P. J. Galante , M. Soley‐Guardia , et al. 2014. “ENMeval: An R Package for Conducting Spatially Independent Evaluations and Estimating Optimal Model Complexity for Maxent Ecological Niche Models.” Methods in Ecology and Evolution 5: 1198–1205.

[ece371741-bib-0055] Oppel, S. , A. Meirinho , I. Ramirez , et al. 2012. “Comparison of Five Modelling Techniques to Predict the Spatial Distribution and Abundance of Seabirds.” Biological Conservation 156: 94–104.

[ece371741-bib-0056] Pardini, R. , A. A. Bueno , T. A. Gardner , P. I. Prado , and J. P. Metzger . 2010. “Beyond the Fragmentation Threshold Hypothesis: Regime Shifts in Biodiversity Across Fragmented Landscapes.” PLoS One 5: 13666. 10.1371/journal.pone.0013666 PMC2965145 21060870

[ece371741-bib-0057] Parmesan, C. 2006. “Ecological and Evolutionary Responses to Recent Climate Change.” Annual Review of Ecology, Evolution, and Systematics 37: 637–669.

[ece371741-bib-0058] Pascual‐Hortal, L. , and S. Saura . 2006. “Comparison and Development of New Graph‐Based Landscape Connectivity Indices: Towards the Priorization of Habitat Patches and Corridors for Conservation.” Landscape Ecology 21: 959–967.

[ece371741-bib-0059] Peterson, A. T. 2003. “Predicting the Geography of Species' Invasions via Ecological Niche Modeling.” Quarterly Review of Biology 78: 419–433. 14737826 10.1086/378926

[ece371741-bib-0060] Phillips, S. J. , R. P. Anderson , and R. E. Schapire . 2006. “Maximum Entropy Modeling of Species Geographic Distributions.” Ecological Modelling 190: 231–259.

[ece371741-bib-0061] Pinheiro, L. C. , Y. O. da Cunha Bitar , U. Galatti , S. Neckel‐Oliveira , and M. C. dos Santos‐Costa . 2012. “Amphibians From Southeastern State of Pará: Carajás Region, Northern Brazil.” Check List 8: 693–702.

[ece371741-bib-0062] R Core Team . 2022. R: A Language and Environment for Statistical Computing. R Foundation for Statistical Computing. http://www.R‐project.org/.

[ece371741-bib-0063] Ribeiro, M. C. , J. P. Metzger , A. C. Martensen , F. J. Ponzoni , and M. M. Hirota . 2009. “The Brazilian Atlantic Forest: How Much Is Left, and How Is the Remaining Forest Distributed? Implications for Conservation.” Biological Conservation 142: 1141–1153.

[ece371741-bib-0064] Riva, F. , and L. Fahrig . 2022. “The Disproportionately High Value of Small Patches for Biodiversity Conservation.” Conservation Letters 15, no. 3: e12881.

[ece371741-bib-0065] Rudnick, D. , S. J. Ryan , P. Beier , et al. 2012. The Role of Landscape Connectivity in Planning and Implementing Conservation and Restoration Priorities. Issues in Ecology. Ecological Society of America.

[ece371741-bib-0066] Sampaio, G. , L. S. Borma , M. Cardoso , A. L. Muniz , C. von Randow , and R. D. Andrés . 2018. “Chapter 8: Assessing the Possible Impacts of a 40°C or Higher Warming in Amazonia.” In Climate Change Risks in Brazil, edited by C. A. Nobre , J. A. Marengo , and W. R. Soares , 201–218. Springer.

[ece371741-bib-0067] Saura, S. , and L. Pascual‐Hortal . 2007. “A New Habitat Availability Index to Integrate Connectivity in Landscape Conservation Planning: Comparison With Existing Indices and Application to a Case Study.” Landscape and Urban Planning 83: 91–103.

[ece371741-bib-0068] Saura, S. , and L. Rubio . 2010. “A Common Currency for the Different Ways in Which Patches and Links Can Contribute to Habitat Availability and Connectivity in the Landscape.” Ecography 33: 523–537.

[ece371741-bib-0069] Saura, S. , and J. Torné . 2009. “Conefor Sensinode 2.2: A Software Package for Quantifying the Importance of Habitat Patches for Landscape Connectivity.” Environmental Modelling & Software 24: 135–139.

[ece371741-bib-0070] Saura, S. , and J. Torné . 2012. Conefor 2.6 User Manual (April 2012). Univ. Politécnica Madrid. www.conefor.org.

[ece371741-bib-0071] Schapire, R. E. 2001. “The Boosting Approach to Machine Learning an Overview.” In Nonlinear Estimation and Classification, edited by D. D. Denison , M. H. Hansen , C. C. Holmes , B. Mallick , and B. Yu . Springer.

[ece371741-bib-0072] Shafer, C. L. 1995. “Values and Short Comings of Small Reserves: Dealing With the Smallest Habitat Fragments When Some of Them Are All That Is Left.” Bioscience 45, no. 2: 80–88.

[ece371741-bib-0085] Smith, M. A. , and D. M. Green . 2005. “Dispersal and the Metapopulation Paradigm in Amphibian Ecology and Conservation: Are All Amphibian Populations Metapopulations?” Echography 28: 110–128.

[ece371741-bib-0073] Soares‐Filho, B. , and R. Rajão . 2018. “Traditional Conservation Strategies Still the Best Option.” Nature Sustainability 1: 608–610.

[ece371741-bib-0074] Souza, C. M., Jr. , J. Z. Shimbo , M. R. Rosa , et al. 2020. “Reconstructing Three Decades of Land Use and Land Cover Changes in Brazilian Biomes With Landsat Archive and Earth Engine.” Remote Sensing 12: 2735.

[ece371741-bib-0075] Swift, T. L. , and S. J. Hannon . 2010. “Critical Thresholds Associated With Habitat Loss: A Review of the Concepts, Evidence, and Applications.” Biological Reviews 85: 35–53. 19930172 10.1111/j.1469-185X.2009.00093.x

[ece371741-bib-0076] Teixeira, C. C. , L. C. Trevelin , M. C. dos Santos‐Costa , A. Prudente , and D. P. Silva . 2022. “Synergistic Effects of Climate and Landscape Change on the Conservation of Amazonian Lizards.” PeerJ 10: 13028. 10.7717/peerj.13028 PMC8973465 35368330

[ece371741-bib-0077] Tischendorf, L. , and L. Fahrig . 2000. “On the Usage and Measurement of Landscape Connectivity.” Oikos 90: 7–19.

[ece371741-bib-0078] Toms, J. D. , and M. L. Lesperance . 2003. “Piecewise Regression: A Tool for Identifying Ecological Thresholds.” Ecology 84: 2034–2041.

[ece371741-bib-0079] Tylianakis, J. M. , R. K. Didham , J. Bascompte , and D. A. Wardle . 2008. “Global Change and Species Interactions in Terrestrial Ecosystems.” Ecology Letters 11: 1351–1363. 19062363 10.1111/j.1461-0248.2008.01250.x

[ece371741-bib-0080] UNEP‐WCMC and IUCN . 2023. Protected Planet: The World Database on Protected Areas (WDPA) and World Database on Other Effective Area‐Based Conservation Measures (WD‐OECM). UNEP‐WCMC and IUCN. www.protectedplanet.net.

[ece371741-bib-0081] Urban, D. , and T. Keitt . 2001. “Landscape Connectivity: A Graph‐Theoretic.” Ecology 82: 1205–1218.

[ece371741-bib-0082] Velazco, S. J. E. , F. Galvão , F. Villalobos , and P. De Marco Júnior . 2017. “Using Worldwide Edaphic Data to Model Plant Species Niches: An Assessment at a Continental Extent.” PLoS One 12: 0186025. 10.1371/journal.pone.0186025 PMC5648144 29049298

[ece371741-bib-0083] Viana, P. L. , N. F. O. Mota , A. S. B. Gil , et al. 2016. “Flora of the Cangas of the Serra dos Carajás, Pará, Brazil: History Study Area and Methodology.” Rodriguésia 67: 1107–1124.

[ece371741-bib-0084] Wickham, H. 2016. Getting Started With ggplot2. ggplot2. Use R! Springer. 10.1007/978-3-319-24277-4_2.

