# Supplementary material for: Assessing Connectivity Thresholds Under Habitat Loss Scenarios for Threatened Amphibians and Squamate Reptiles in the Eastern Brazilian Amazon
Source: Ecol Evol. 2025 Jul 15;15(7):e71741. doi: 10.1002/ece3.71741 (PMC12260477; doi:10.1002/ece3.71741)
Supplement: Supplementary file 1 — Data S1. [file ECE3-15-e71741-s001.docx]

**SUPPLEMENTARY MATERIAL 1 – FIGURES AND CODE**

**Assessing connectivity thresholds under habitat loss scenarios for threatened amphibians and squamate reptiles in the eastern Brazilian Amazon**

Cássia Teixeira^1,2^, Gisele Lopes Nunes^2^, Leonardo Carreira Trevelin^1,2^, Daniel Paiva Silva^3^, Ana Lúcia da Costa Prudente^1^

^1^ Programa de Pós-Graduação em Biodiversidade e Evolução, Museu Paraense Emílio Goeldi, Belém, Pará, Brazil

^2^ Instituto Tecnológico Vale – Desenvolvimento Sustentável, Belém, Pará, Brazil

^3^ COBIMA Lab, Departamento de Biologia, Instituto Federal Goiano – Campos Urutaí, Goiás, Brazil

Corresponding authors: leonardo.trevelin@itv.org /cassiacteixeira@gmail.com

This document includes:

**Figure S1.** **Maps of habitat areas for each species.**

**Figure S2. Scenarios of habitat areas in the landscape for one species.**

**Figure S3. Maps of habitat areas with at least 30% habitat amount for each species.**

**Code S1. Script for SDMs** **in R environment.**

**Code S2. Script for Piecewise regression analysis in R environment.**

Supplementary tables are in a separate file: Supplementary Material 2 - Tables.


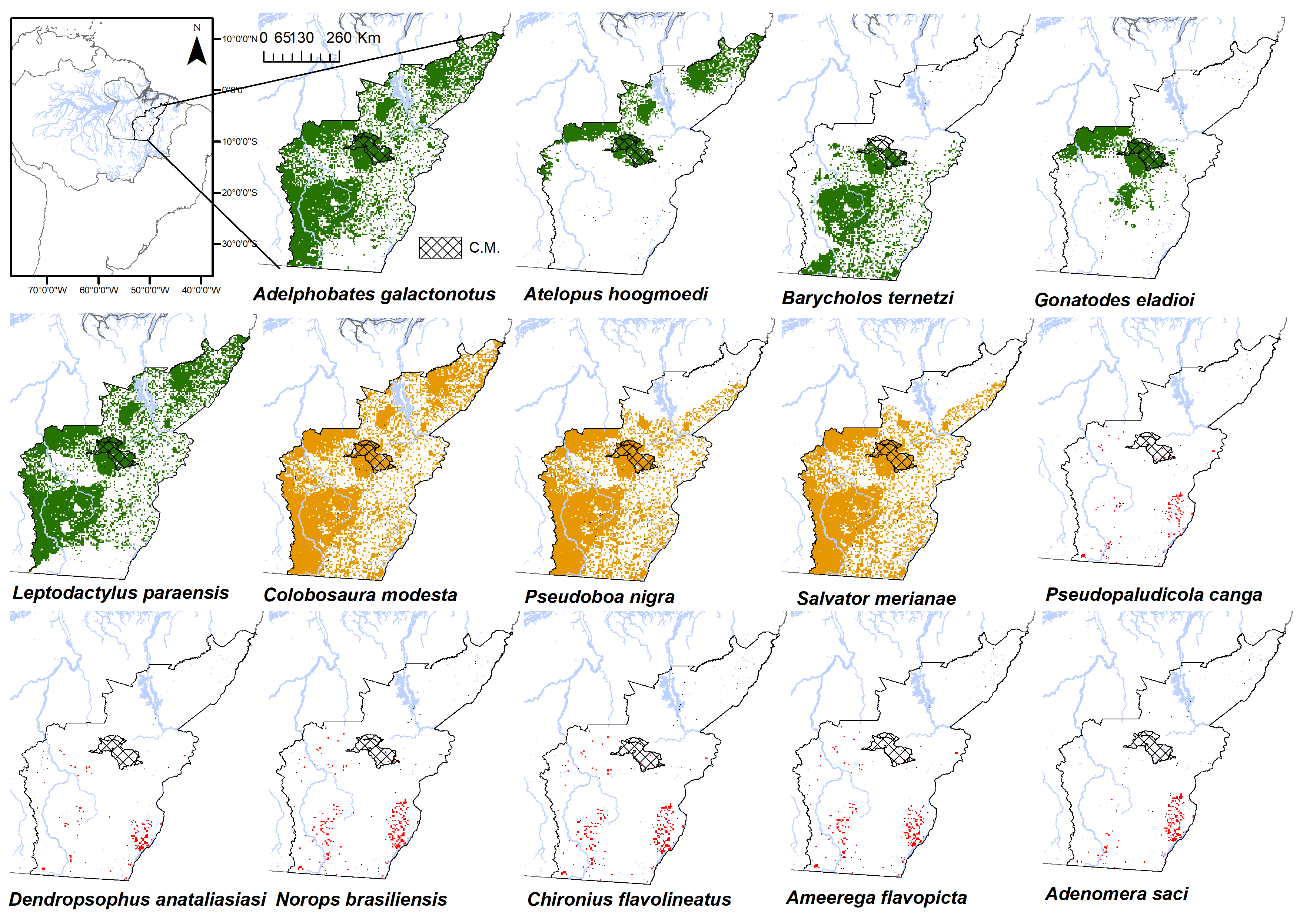


**Figure S1.** Maps of habitat areas (climate models superimposed with the vegetation layer) predicted for species of interest for conservation in Southeastern Pará. Green: habitat areas for forest species. Yellow: habitat areas for forest and canga species. Red: habitat areas for canga species. CM: Carajás Mosaic.


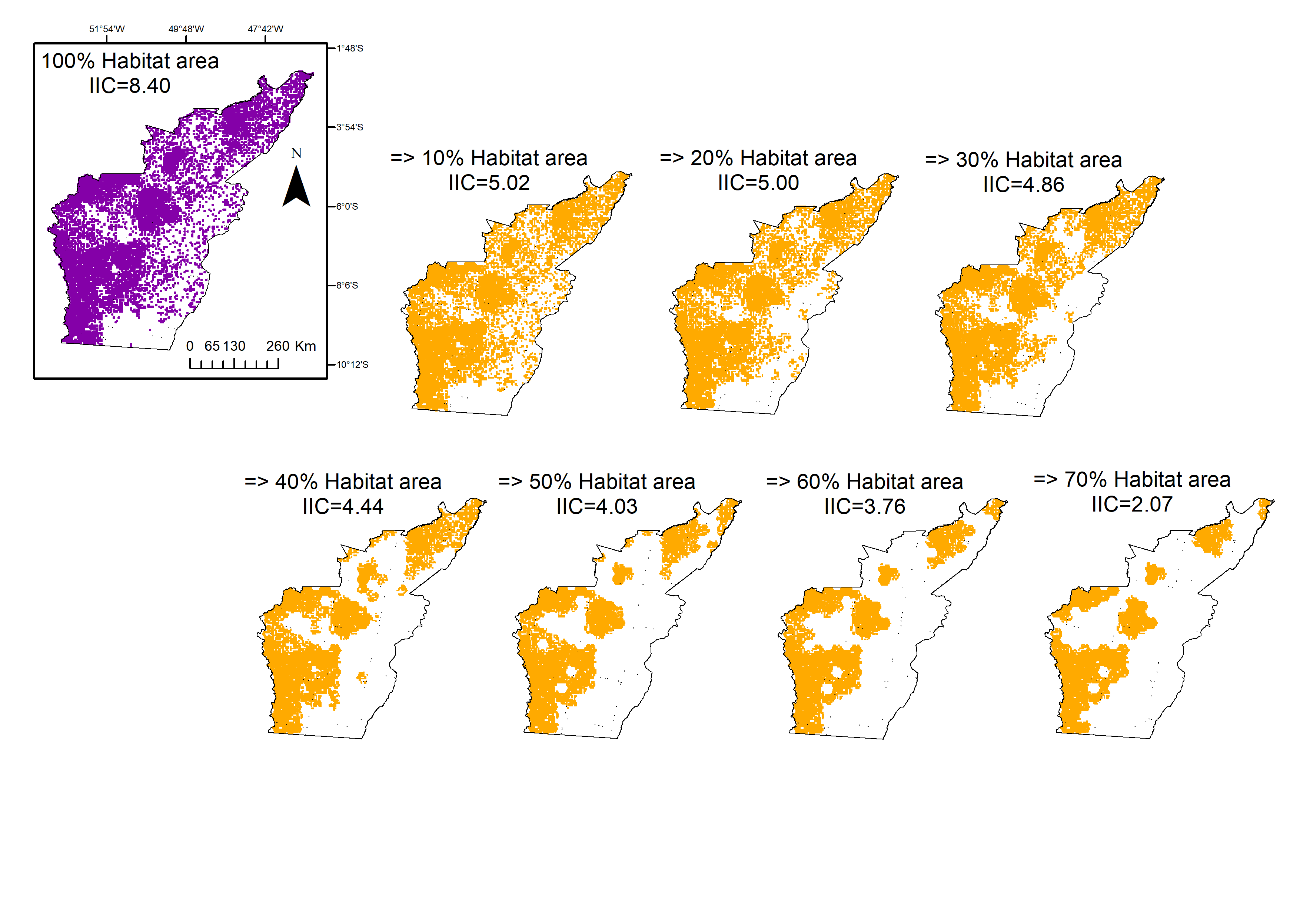


**Figure S2**. Scenarios of habitat area maps for the anuran *Adelphobates galactonotus*. The purple map indicates the map with the entire available habitat area, and the yellow maps indicate the evaluated landscape removal scenarios. The total distribution in Southeast Pará decreases as landscapes with less habitat are removed, and this pattern was repeated for the other species. The Integral Connectivity Index (IIC) values ​​are presented in each scenario map.


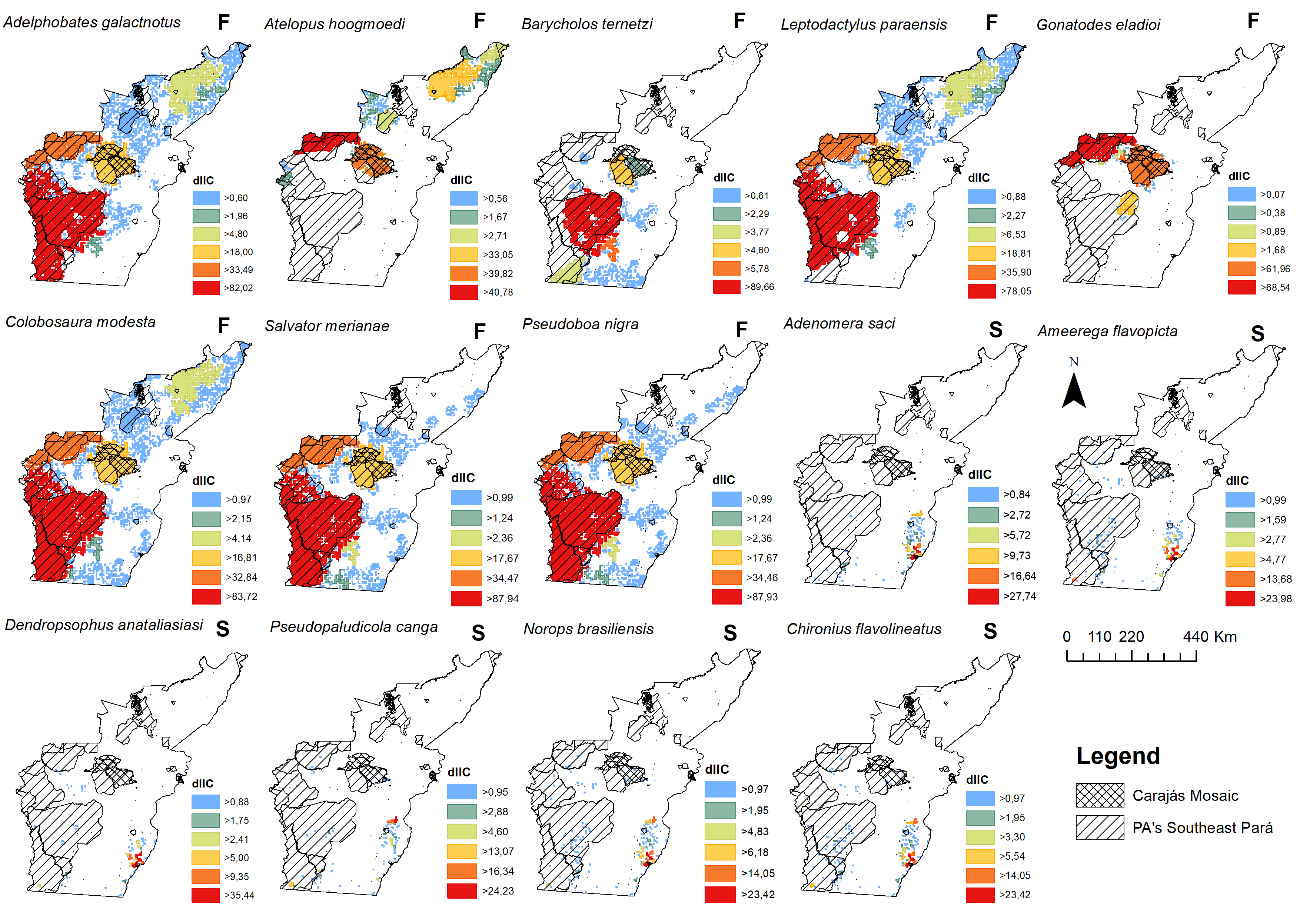


**Figure S3**. Maps showing habitat areas with at least 30% habitat amount for forest species (F) and with 100% habitat amount for canga species (S). These areas are classified according to the importance of the patches, indicated by the dIIC. Cooler colors indicate less importance and warmer colors indicate more importance of patches for maintaining connectivity.

**CODE S1**. Script for SDMs in R environment.

# SDMs procedures for generating potential distribution maps.

# Obtained from the ENMTML package (<https://github.com/andrefaa/ENMTML>).

# Andrade et al. (2020).

# 0- Load libraries ####

library(ENMTML)

library(devtools)

library(rgdal)

library(rgeos)

# 1- Load data and SDMs parameters ####

ENMTML(pred_dir = "C:/model_24/ambientais", # environmental variables

occ_file="C:/model_24/ocorrencias_amazonia.txt", # occurences file

result_dir="C:/model_24/results", #output directory

proj_dir= NULL, #no projections

sp="species", # species column name

x="long", # coordinates column name

y="lat", # coordinates column name

min_occ=9, # minimum occurrences

thin_occ=c(method='CELLSIZE'), # spatial filtering

colin_var = c(method='PCA'), # method to reduce variable collinearity

imp_var = FALSE, # not used

eval_occ = NULL, # not used

sp_accessible_area = c(method='MASK', filepath='C:/model_24/shape_as/America_do_sul.shp'), # extension of SDMs

pres_abs_ratio = 1, # presence-absence ratio

pseudoabs_method = c(method='ENV_CONST'), # pseudo-absence allocation method

part = c(method= 'BLOCK'), # partition method for model’s validation

save_part=FALSE,save_final=TRUE, # save partial and final models

algorithm = c("MXS","RDF","SVM"), # algorithms

thr=c(type=' MAX_TSS'), # threshold used for presence-absence predictions

msdm=NULL, # not used

ensemble = c(method='MEAN'), # ensemble method

extrapolation=FALSE, # not used

cores=1) # default

**CODE S2**. Piecewise Regression for critical thresholds in landscape connectivity ~ habitat amount

# Cassia Teixeira e Leonardo Trevelin

# jun/2025

# Load library ####

library(ggplot2)

library(segmented)

# Load data ####

cassia <- read.csv("IIC_Final_2.csv", head=TRUE, sep = ";", dec = ".", stringsAsFactors = T)

str(cassia)

names(cassia)

cassia_1000 <- subset(cassia, Sensibility == 1000) # subset dataset for different dispersal distances

cassia_500 <- subset(cassia, Sensibility == 500)

cassia_1500 <- subset(cassia, Sensibility == 1500)

# Visualize data ####

ggplot(cassia_1000, aes(x = Quant_Habit, y=IIC, group = Sensibility))+

geom_point(aes(colour=Habitat, shape=Taxon), position = "jitter")+

scale_colour_manual(values=c("green", "blue","red"), labels = c('Forests and Savannas', 'Forests', 'Savannas'))+

labs(y="Integral Index of Connectivity (IIC)", x= "Minimum amount of habitat per landscape (%)")+

theme_minimal()+

theme(axis.title.x = element_text(size = 14),

axis.title.y = element_text(size = 14),

legend.text = element_text(size = 14))

# Modeling the complete dataset (using 1000m dispersal distances) ####

str(cassia_1000)

fit_compl <- lm(IIC ~ Quant_Habit, data=cassia_1000) #Fit a linear regression model

summary(fit_compl)

segmented.fit_compl <- segmented(fit_compl, seg.Z = ~Quant_Habit, psi=40) # fit a segmented regression model

# Note we are suggesting a starting breakpoint of 40-50% based on data visualization

summary(segmented.fit_compl)

segmented.fit_compl2 <- segmented(fit_compl, seg.Z = ~Quant_Habit, npsi=2) # Fit a second segmented regression model

# now we just suggest there are two breakpoints to the function

summary(segmented.fit_compl2) #checar o resultado

# Select most parsimonious model

anova(fit_compl, segmented.fit_compl, segmented.fit_compl2) # Loglikehood Ratio Test

AIC(fit_compl, segmented.fit_compl, segmented.fit_compl2) # Using Akaike (AIC)

segmented.fit_compl # selected model smaller AIC

summary(segmented.fit_compl)

# Model species from specifc Habitats (using 1000m dispersal distances) ####

# Subset data just for Forest-exclusive and Forest-Savana species

cassia_Flor_FlorSav <-subset(cassia_1000, Habitat == "Forests and Savannas" | Habitat == "Forests")

fit_compl <- lm(IIC ~ Quant_Habit, data=cassia_Flor_FlorSav) #LM

summary(fit_compl)

segmented.fit_compl <- segmented(fit_compl, seg.Z = ~Quant_Habit, npsi=1) #segmented-LM

summary(segmented.fit_compl)

segmented.fit_compl2 <- segmented(fit_compl, seg.Z = ~Quant_Habit, npsi=2) # segmented-LM 2

summary(segmented.fit_compl2)

# Select most parsimonious model

anova(fit_compl, segmented.fit_compl, segmented.fit_compl2) # Loglikehood Ratio Test

AIC(fit_compl, segmented.fit_compl, segmented.fit_compl2) # Using Akaike (AIC)

segmented.fit_compl # selected model smaller AIC

summary(segmented.fit_compl)

# 95% Confidence intervals

psilower = 32.027 - 1.96 * 6.56

psiupper = 32.027 + 1.96 * 6.56

# Predict new values for for graph

x_novo <- seq(min(cassia_Flor_FlorSav$Quant_Habit), max(cassia_Flor_FlorSav$Quant_Habit), length.out = 200)

# Data frame for prediction results

dados_pred <- data.frame(

Quant_Habit = x_novo

)

# predictions

dados_pred$fit <- predict(segmented.fit_compl, newdata = dados_pred, type = "response")

# confidence intervals predictions in link scale, than transform if needed (not the case here)

pred_link <- predict(segmented.fit_compl, newdata = dados_pred, se.fit = TRUE, type = "link")

#output for graph

dados_pred <- dados_pred %>%

mutate(

fit_link = pred_link$fit,

se_link = pred_link$se.fit,

lower = fit_link - 1.96 * se_link,

upper = fit_link + 1.96 * se_link,

fit = fit_link

)

# Final graphical output ####

ggplot() +

geom_point(data = cassia_1000, aes(x = Quant_Habit, y = IIC, colour=Habitat, shape=Taxon) , alpha = 0.8, position = "jitter") +

scale_colour_manual(values=c( "#d95f02", "#1b9e77", "#e7298a") , labels = c('Forests and Savannas', 'Forests', 'Savannas')) +

geom_ribbon(data = dados_pred, aes(x = Quant_Habit, ymin = lower, ymax = upper),

fill = "#7570b3", alpha = 0.2) +

geom_line(data = dados_pred, aes(x = Quant_Habit, y = fit), color = "#7570b3", size = 1) +

geom_segment(aes(x = 32, xend = 32, y = 4.5, yend = 6), color = "black", linetype = "dashed", size = 0.5)+

geom_errorbarh(aes(y=5.3,xmin = 19.17, xmax = 44.88), height = 0.2, color = "black", alpha = 0.6) +

annotate("text", x = 42.5 , y = 5.8, label = "Breakpoint = 32 (19-44)", size = 3.5, color = "black")+

labs(

x = "Minimum amount of habitat in the landscape (%)",

y = "Integral Index of Connectivity (IIC)",

) +

theme_minimal() +

theme(

axis.title.x = element_text(size = 14, face = "bold"),

axis.text.x = element_text(size = 12),

axis.title.y = element_text(size = 14, face = "bold"),

axis.text.y = element_text(size = 12),

legend.text = element_text(size = 12),

legend.position = c(1, 1), # canto superior direito

legend.justification = c(1, 1), # ancora o canto superior direito da legenda

legend.background = element_rect(fill = "white", color = NA)

)

ggsave("Figure3.jpg", device = "jpeg", dpi="print", units="cm", width = 18, height=15)
